# Seeing an Unseen Science

**DOI:** 10.1371/journal.pbio.1001659

**Published:** 2013-10-01

**Authors:** Karl S. Matlin

**Affiliations:** Department of Surgery, The University of Chicago, Chicago, Illinois, United States of America

## Abstract

Karl Matlin explores the origins of cell biology in this review of Carol Moberg's *Entering an Unseen World.*

[Fig pbio-1001659-g001]The almost complete omission of cell biology from the history of modern biology is perplexing. For instance, Allen published his well-regarded *Life Science in the Twentieth Century* in 1975, the year after the founders of modern cell biology won the Nobel Prize, yet his book does not mention cell biology [1]. Judson's massive hagiography *The Eighth Day of Creation* (1979), which tracks the development of molecular biology, also leaves out cell biology, referring to George Palade, one of the recipients of the Nobel Prize, on only three pages [2]. Hopefully, with the appearance of *Entering an Unseen World* [3], reviewed here, a more accurate historical balance will begin to be restored.

**Figure pbio-1001659-g001:**
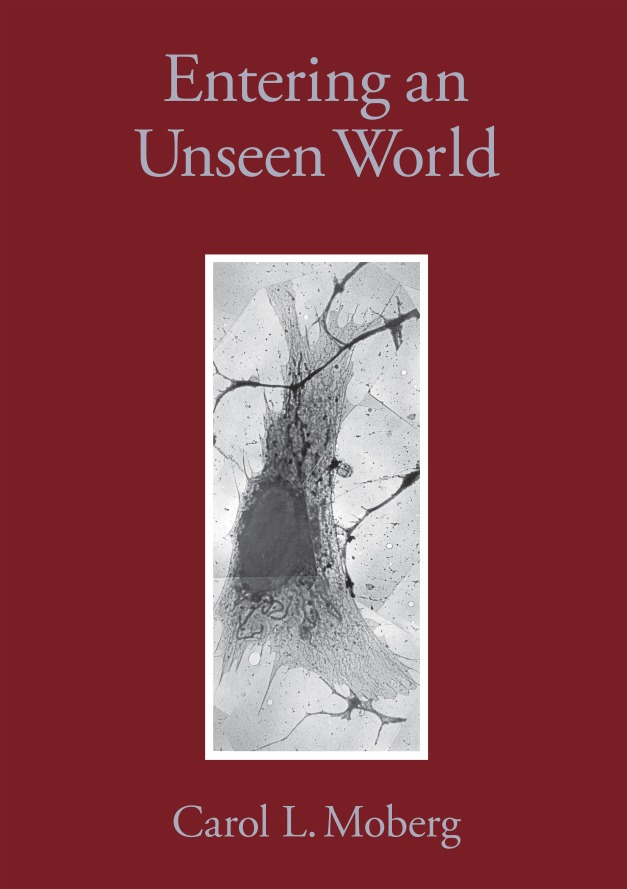
Moberg CL (2012) Entering an Unseen World. New York: Rockefeller University Press. 499 p. ISBN 978-0874700633 (hardcover). US$40.00.

Why has cell biology been almost ignored? One possible reason might be that the gene-centric view of biology, which arose after the rediscovery of Mendel at the beginning of the twentieth century and reached its apotheosis with elucidation of the DNA structure in 1953, crowded out other perspectives. Molecular biology did not reach its position of prominence in the history of biology solely on the basis of its achievements. Indeed, the version of molecular biology promoting extreme reductionism as an explanatory strategy began to decline by the mid-1960s [4]. About that time, molecular biology, diminished as a worldview, was reincarnated as a set of very powerful and useful technologies, all focused on manipulation of DNA. Almost simultaneously, the reputation of molecular biology as the pinnacle of late twentieth century science was also boosted by a pervasive public relations campaign. An early example was Watson's *The Double Helix*, followed by Judson's modestly titled book [2],[5]. Later articles and press releases from molecular biologists in the run up to completing the human genome sequence eclipsed the earlier efforts [6]. These statements promoted genomic DNA as the “code of codes” whose sequence might even solve homelessness [6],[7]. While today the air may have come out of such inflated arguments, reductive molecular biological approaches continue to be touted as key elements of “big data” strategies for explaining everything biological.

In addition to the molecular biology media fest, another factor contributing to the low profile of cell biology in the history of twentieth century science may be the paucity of scholarship focused on its origins. Bechtel's excellent *Discovering Cell Mechanisms*—relating cell biology's development until 1970—is a notable exception, as is Rasmussen's *Picture Control*, an account of electron microscopy applied to biology [8],[9]. Even Rheinberger's *Toward a History of Epistemic Things*, which concentrates on early studies of protein synthesis, highlights the crucial roles of cell biology and biochemistry in discoveries that are commonly claimed as achievements of molecular biology [10]. Now, with the publication of Carol Moberg's *Entering an Unseen World*, an additional valuable contribution has arrived [3].

Focusing on the years from 1910 to 1974, Moberg's book maps the origins of modern cell biology as it developed at the Rockefeller Institute (later University) in New York City. Divided into three chronological sections, the book is a hybrid of original chapters written by Moberg and based upon her extensive archival research, and other chapters contributed as historical essays by actual practitioners of the science. The late Philip Siekevitz, himself a major figure in the history of cell biology, also wrote or cowrote several contributions not directly related to his own work. Moberg's chapters, which focus on the early period between 1910 and 1949, relate how Peyton Rous' work on a chicken sarcoma led to the establishment of a cancer research laboratory ultimately headed by James Murphy. Murphy hired Albert Claude, and subsequently brought in Keith Porter and then George Palade. Claude developed differential centrifugation, initially with the goal of purifying the tumor agent from chickens, but later used the approach to separate normal cellular components. Motivated by Claude, Porter developed methods that made it possible to view intact mammalian cells in the electron microscope. Palade then merged these technologies into a powerful investigational approach that ultimately established cell biology as a distinct discipline.

Moberg's thesis is that cell biology evolved from cancer research at Rockefeller. Although it is certainly true that the laboratory of cancer research provided a home for the investigators who would eventually create cell biology, to say that cell biology was an outgrowth of cancer research is like saying that a plant is an outgrowth of the soil in which it was planted. The laboratory of cancer research provided the conditions and nourishment for cell biology, but cell biology was its own thing. Moberg supports her thesis with a quote from Porter's 1939 letter to Murphy inquiring about a position: “It has occurred to me that an embryologist [as he was] should be well fitted to study tumour development” [3, p. 44]. However, Porter's letter can be interpreted more as an attempt to please a potential employer at the end of the job-starved Depression than the statements of a committed cancer biologist. Indeed, Moberg's description of the various research initiatives of the Murphy lab makes it clear that the work on cancer went nowhere. As soon as Porter and Palade had the opportunity to follow their own interests after Claude returned to Belgium and Murphy retired in 1950, they never worked directly on cancer again.

It seems more reasonable to place the origins of modern cell biology within a larger historical context. In the nineteenth century, improvements in light microscopy helped to establish cells as the fundamental units of living organisms. By the end of the century, cytology had become an experimental rather than a purely morphological science. Its greatest achievement was the chromosomal theory of heredity that, when linked with Mendel's work, gave rise to genetics. A monumental 1924 text authored by the most prominent group of cytologists at the time celebrated the field [11]. At the end of the introduction to this work, E. B. Wilson states:

[E]arlier morphological cytology has broadened into a many-sided *cellular biology*…in which observation and experiment, morphology and physiology, have entered into close affiliation with one another and with biophysics and biochemistry [11, p. 10, italics original].

The promise of this prediction, however, went unfulfilled for want of appropriate techniques, and cytology stagnated. Even though there was great interest in delving into the chemical and physical nature of cellular function, the resolution of light microscopy was limited by diffraction and methods to explore cells at the molecular level were ineffective.

This began to change when Claude developed cell fractionation and realized that the particles that he partially purified and biochemically characterized in tumor cells were also in normal cells [8]. In 1945, when Porter finally worked out how to see whole cultured cells in the electron microscope, both Claude and Porter realized that another major limitation to the advance of “cellular biology” (à la Wilson) had been overcome [12]. Moberg quotes an unpublished 1970 lecture by Porter describing his reaction to seeing the first cell in the electron microscope:

It was wonderful, believe me, we had never seen anything like it. Men have visited the moon…but we were the first…to see particles, to see structures that the light microscope had not been able to resolve [cited in 3, p. 60].

Even Claude, whose interest in cancer had led him to Rockefeller, understood that his most important discoveries aligned more closely with the long history of fundamental cell studies than with a particular disease. He begins his 1948 Harvey Lecture by recounting the history of light microscopy, and then goes on to describe his work on normal cells with almost no mention of cancer [13].

Even though Moberg's book advances a questionable view of cell biology's genealogy, it remains an invaluable resource for future studies. Although past work on cell biology has made use of interviews and archives, nobody has delved deeper than Moberg. In particular, Moberg gained access to Albert Claude's private papers, and the letters she found there were particularly insightful. Furthermore, her extensive examination of James Murphy's papers thoroughly illuminates the inner workings of the laboratory of cancer research in its early years. The essays provided by Rockefeller scientists, however, while intrinsically interesting as historical artifacts in themselves, are uneven in their quality. Moberg is forced, in some cases, to resort to excerpts from previously published memoirs to fill gaps in the chronology. Nevertheless, many are stories that have never been told, such as Marilyn Farquhar's description of how the discovery of tight junctions came about [3]. Moberg laudably takes a broad view of cell biology to tell her story. Her long relationship with Zanvil Cohn's laboratory of cellular immunology at Rockefeller enables her to describe how studies of phagocytes provided insight into fundamental mechanisms of endocytosis [3]. This part of the book is capped by Ralph Steinman's essay on endocytosis and his discovery of dendritic cells, a story fortunately captured before his untimely death [3, pp. 335–346].

Some might argue that the focus of the book on Rockefeller distorts history, but this is not the case: Moberg does not limit her analysis to contributions from Rockefeller scientists. Even so, when one finishes *Entering an Unseen World*, one has to conclude that, to an amazing extent, Rockefeller *was* the wellspring of modern cell biology. Its scientists not only developed key technologies that enabled them to investigate cells at the molecular level, but they were also instrumental in founding the American Society for Cell Biology and the *Journal of Cell Biology*. Most importantly, the Rockefeller group devised a powerful explanatory approach to link cellular structure and function. Claude specifically refers to this in his 1948 Harvey Lecture when he states: “[I]t would be difficult to separate the biochemical work from the morphological observations since the [electron] microscope has constantly served as a guide or check for the chemical and biochemical studies” [13, p. 123]. Today this strategy lives on every time a fluorescent protein is tracked through a living cell. The form of the cell—its morphology—provides the context that makes molecular manipulations meaningful in a biologically significant way. Perhaps, if *Entering an Unseen World* leads to a reexamination of the history of modern biology, the still powerful contextual and iterative approach devised by cell biologists to understand complex biological systems will finally receive the credit for which it is long overdue.

**About the Author**

Karl Matlin is a cell biologist whose research has focused on mechanisms of epithelial cell polarization. He obtained his PhD from the Rockefeller University in 1979, where he worked in the Laboratory of Cell Biology. Currently, he is Professor and Vice-Chair for Research at the University of Chicago Department of Surgery, and he is writing a book on the history of molecular cell biology from the perspective of the Signal Hypothesis.
